# Effects of typical emerging pollutants on biological treatment systems: processes, mechanisms, and recovery strategies

**DOI:** 10.3389/fmicb.2026.1998868

**Published:** 2026-10-07

**Authors:** Xiaoying Ren, Aiwen Song, Da Shang, Huai Li

**Affiliations:** 1 Northeast Institute of Geography and Agroecology, Chinese Academy of Sciences, Changchun, China; 2 University of Chinese Academy of Sciences, Beijing, China

**Keywords:** influential processes, microplastics, nanoparticles, perfluoroalkyl and polyfluoroalkyl substances, recovery strategies

## Abstract

With the rapid advancement of industrialization and urbanization, human activities have released large quantities of persistent emerging contaminants into the environment. Among these, nanomaterials, microplastics, and perfluoroalkyl and polyfluoroalkyl substances have emerged as research hotspots due to their widespread applications and increasingly significant environmental risks. These contaminants exhibit strong environmental persistence in aquatic environments and significantly affect the stability and treatment efficiency of conventional biological treatment systems, posing new challenges to the normal operation and pollutant removal. This article reviews the migration and transformation behavior of these emerging contaminants in biological treatment systems, the processes and mechanisms underlying their impacts on microbial communities and metabolic activities, as well as recovery strategies. By summarizing existing research results, this article aims to provide a scientific basis and technical support for the process optimization and the development of pollutant reduction technologies in wastewater treatment systems.

## 1 Introduction

Emerging contaminants (ECs) refer to chemicals that have not been systematically monitored but may pose risks to the environment and biological health. They include nanomaterials, microplastics and per- and polyfluoroalkyl substances (PFAS) (Khan et al., 2023). Due to the increasing prominence of their complex molecular structures, environmental persistence, and potential ecological hazards, these contaminants have received increasing attention. Among them, nanomaterials have attracted considerable attention because of their large surface areas and unique reactivity. These characteristics have contributed to their widespread use in industrial manufacturing and various consumer goods, including electronic devices, healthcare materials and cosmetics (Demirel, 2016). However, once released into the environment, nanomaterials may exert toxic effects on microbial communities and destroy the ecosystem balance (Sun et al., 2022). Microplastics, owing to their small particle size (typically less than 5 millimeters), high adsorption capacity, and bioavailability, can act as pollutant carriers in aquatic ecosystems and potentially amplify contaminant toxicity (Habumugisha et al., 2024). PFAS due to its stable fluorocarbon bond structure and pronounced hydrophobic and oleophobic properties, are difficult to remove during wastewater treatment and may can cause long-term damage to key microbial communities (Amen et al., 2023).

The unique physicochemical properties and environmental behaviors of these typical contaminants make their impact on biological treatment systems an important issue requiring in-depth research. Biological treatment is a major technology for treating conventional pollutants. However, the widespread occurrence and release of emerging contaminants (ECs) pose new challenges to the stability and efficiency of biological treatment systems. The introduction of ECs may negatively affect microbial activity, metabolism, and community structure, thereby impairing the overall performance of wastewater treatment systems (Shi K. et al., 2024). In addition, these contaminants typically occur at low concentrations and in mixed states, and their interactions with coexisting pollutants may be synergistic or antagonistic, further increasing the complexity of risk assessment (Yu et al., 2024).

In recent years, numerous studies have revealed the interactions and impacts of nanomaterials, microplastics, and PFAS on biological treatment processes. Nanomaterials may inhibit microbial activity through oxidative stress (Yusuf et al., 2021; Olawade et al., 2024); microplastics may disturb biochemical processes within the reactors through physical interference and chemical adsorption (Huang et al., 2024); and PFAS may inhibit pollutant removal by altering microbial gene expression or disrupting cell membrane function (Naumann et al., 2022). Existing studies have also shown that these emerging contaminants can alter the operational properties of wastewater treatment systems, such as sludge settleability and mass transfer efficiency, and affect microbial functions through pathways including oxidative stress, membrane damage, and gene regulation. Nevertheless, Several key problems remain. (1) Unclear mechanisms. The mechanisms underlying the toxic effects of different contaminants and corresponding microbial responses have not been systematically elucidated, particularly under combined exposure conditions; (2) insufficient quantification. Quantitative relationships among environmental concentrations, effect doses, microbial responses, and treatment performance remain insufficiently established. Many studies have focused on relatively high exposure concentrations, whereas these contaminants typically occur at much lower concentrations in real environments; (3) Lack of recovery and regulation strategies. Although biological treatment systems have been used to cope with emerging contaminants, their economy and long-term feasibility are still to be verified, especially the application effect in practical engineering is still unstable, and long-term operation may face problems such as inactivation of bacterial communities. Therefore, the processes, mechanisms, and recovery strategies associated with emerging contaminants in biological treatment systems require further investigation.

The present review has four main objectives: (1) To synthesize the impacts of nanomaterials, microplastics, and per- and polyfluoroalkyl substances (PFAS) on biological treatment systems; (2) To elucidate the mechanisms by which these contaminants alter microbial community structure, metabolic activity, and biological treatment performance; (3) To assess the effectiveness of existing recovery strategies and their prospects for technological applications; (4) To identify future research directions and targeted recommendations to address emerging contaminant challenges in biological treatment systems. Through this review, we aim to deepen the conceptual framework for interpreting the interactions between emerging contaminants and biological treatment systems, and to identify scientifically grounded approaches that can facilitate sustainable progress in wastewater treatment technologies.

## 2 Impacts, mechanisms and recovery strategies of nanomaterials on biological treatment systems

The impacts, mechanisms, and recovery strategies of nanomaterials in biological treatment systems represent key research areas in environmental engineering and ecotoxicology. With the rapid advancement of nanotechnology, a wide range of nanomaterial are extensively applied in industrial, medical, and consumer products. These materials eventually enter the environment through wastewater discharges, where they exert complex effects on biological treatment systems.

### 2.1 Impact of nanomaterials on biological treatment systems

#### 2.1.1 Influence of micro-organisms

Nanomaterials can affect microbial growth and metabolic activities through direct contact with microbial cells or ion release (Kedves et al., 2024a). For example, metallic nanoparticles (e.g., Ag, CuO) possess strong antimicrobial properties that may cause mortality or substantial reduction of certain bacterial populations, thereby altering microbial community diversity (Olawade et al., 2024). The introduction of nanomaterials may also result in reduced microbial diversity, particularly through the significant inhibition of key functional taxa (Li et al., 2017; Cervantes-Aviles et al., 2021). One reported case is that excessive CuO nanoparticle exposure disrupts the functioning of methanogenic communities, ultimately lowering methane production during anaerobic digestion (Abdulsada et al., 2025).

#### 2.1.2 Enzyme activity inhibition

Interactions between nanomaterials and vital biomolecules (e.g., dehydrogenases, proteases) can modify or block enzyme active centers, resulting in decreased catalytic activity and reduced biochemical reaction rates. Wang et al. (2021) reported that TiO₂ nanoparticles may inhibit enzymes involved in organic degradation, thereby reducing pollutant removal efficiency. Nanomaterials may also impair enzymatic activity through oxidative stress, thereby further disrupting the metabolic functions of microorganisms. Furthermore, the inhibitory effects of nanomaterials on enzymes are strongly influenced by their surface charge, particle size, and concentration (Wang S. et al., 2020).

#### 2.1.3 Impact on biofilm systems

Nanomaterials can attach to biofilms and modify their physicochemical characteristics, such as reducing permeability or enhancing fragility. Prolonged exposure may eventually cause biofilm detachment or functional decline. Previous studies have demonstrated that the accumulation of nanomaterials can compromise the structural integrity of biofilms, thereby reducing their pollutant removal efficiency (Dakal et al., 2016; Lee and Lee, 2019; Liao et al., 2019). For example, high concentrations of TiO₂ nanoparticles can induce biofilm detachment and consequently reduce the treatment efficiency of biofilm reactors (Kedves et al., 2024b). In addition, nanomaterials may influence the adhesive capacity and stability of biofilms by modifying their surface charge and hydrophobicity.

#### 2.1.4 Inhibition of anaerobic digestion

High concentrations of certain nanomaterials (e.g., ZnO, NiO) can inhibit microbial activity during the acidogenesis and methanogenesis stages, thereby substantially reducing biogas production. As an illustration, high levels of ZnO nanoparticles have been reported to impair methanogenic bacterial activity, thereby diminishing methane generation during anaerobic digestion (Zheng et al., 2015). In addition, nanomaterials may compromise the stability of the overall anaerobic digestion process by impairing microbial activity in the hydrolysis and acidogenesis stages. Findings across multiple studies reveal that nanomaterial-induced toxicity is not intrinsic alone but is modulated by parameters including concentration levels, particle size distributions, and specific surface functionalities (Guo et al., 2019; Wang S. et al., 2020).

#### 2.1.5 Changes in sludge properties

Nanoparticles can affect the flocculation performance and settling characteristics of activated sludge, thereby complicating system operation. Chen et al. (2014) found that the addition of high concentrations of CuNPs to activated sludge significantly reduced sludge surface hydrophobicity, decreased flocculation ability, and made flocs more dispersible. These changes reduced the sludge settling rate and could even trigger sludge bulking (Chen et al., 2014). In addition, the accumulation of nanomaterials may alter the dewatering performance of sludge and further complicate sludge treatment. For example, Ag nanoparticles may inhibit microbial activity in sludge at high concentrations, leading to a significant decrease in sludge settling performance (Alizadeh et al., 2019; Shi H. X. et al., 2024).

### 2.2 Mechanisms of influence of nanomaterials on biological treatment systems

#### 2.2.1 Direct toxicity

The direct toxicity of nanomaterials in biological treatment systems is chiefly associated with their capacity to compromise microbial membranes and induce oxidative stress. Owing to their extensive surface area and pronounced reactivity, nanomaterials readily attach to cell envelopes, where they disturb membrane architecture, reduce structural stability, and trigger the leakage of intracellular materials. These combined effects hinder microbial physiological processes and may culminate in cell death (Table 1). Olawade et al. (2024) reported that nanoparticles—especially those characterized by reactive surfaces or angular morphologies—are capable of penetrating microbial cell envelopes, which results in membrane destabilization and cytoplasmic effusion. Similarly, Qayyum et al. (2017) confirmed membrane rupture and intracellular leakage in green-synthesized AgNP–bacteria interaction experiments, using SEM/TEM micrographs combined with protein and reducing sugar leakage assays. Experimental evidence and reviews further demonstrate that typical metallic nanoparticles (e.g., AgNPs) and photocatalytic nanomaterials (e.g., TiO₂) can damage microbial membranes via multiple mechanisms. First, physical adsorption or mechanical contact allows particles to disrupt cell surfaces directly. Second, chemical processes are involved: AgNPs release Ag^+^ ions that interact with membrane components and inhibit respiratory enzymes, whereas TiO₂ generates reactive oxygen species (ROS) under light irradiation, oxidizing membrane lipids and proteins. These processes increase membrane permeability, promote intracellular leakage, and eventually cause cell death (Qayyum et al., 2017; More et al., 2023; Olawade et al., 2024; Rodrigues et al., 2024; Serov et al., 2024). In particular, AgNPs have the ability to interact with thiol-containing membrane proteins, disrupting their normal function and consequently promoting additional leakage of intracellular materials.

**Table 1:** Effects and mechanisms of various nanomaterials on microbial activity and performance in biological wastewater treatment systems.

Nanomaterial type	Study system/target	Main findings	References
AgNPs	High-rate moving bed biofilm reactor (MBBR)	Reported suppression of biofilm activity at 131 μg·L^−1^ (18 d) and 631 μg·L^−1^ (5 d), leading to decreased organic (COD) removal efficiency — indicating AgNPs inhibit microbial activity and impair treatment performance.	Alizadeh (2019)
Ag NPs	Activated sludge bacteria	Ag NPs directly contact cell membranes, causing membrane rupture and cytoplasmic leakage.	Chen et al. (2014)
Ag NPs (green synthesis)	Bacterial cultures/biofilm experiments (model bacterial biofilms)	SEM/TEM observations show membrane damage; intracellular proteins and reducing sugars leak out. AgNPs induce ROS generation, membrane rupture, and leakage of intracellular contents.	Qayyum et al. (2017)
TiO₂ NPs	MBR/Activated sludge (membrane bioreactor)	Suspended TiO₂ alters physical properties of sludge flocs (size distribution, EPS), increases cell permeability, and affects membrane filtration/floc stability — supporting that TiO₂ can damage microorganisms and floc structures by chemical/physical means.	Chandoliya et al. (2024)
Ag NPs, TiO₂ NPs	Engineered wastewater treatment processes (attached-growth/moving-bed)	AgNPs and TiO₂ undergo physicochemical transformations during treatment; Ag can accumulate and release ionic Ag (ecotoxicologically relevant), while TiO₂ is relatively stable but can alter attached-growth community composition.	Steinhoff et al. (2020)
ZnO NPs	Anaerobic digestion (waste activated sludge/anaerobic reactors)	ZnO exhibits dose-dependent toxicity in anaerobic digestion: high doses significantly suppress methane production and inhibit key enzyme activities; mechanisms largely attributed to Zn^2+^ dissolution and disruption of microbial community structure.	Vempati and Kumar (2024)
Fe₃O₄ NPs	Aerobic/anaerobic activated sludge systems	High concentrations of Fe₃O₄ lead to increased ROS and LDH release (indicator of cytoplasmic leakage), inhibit key enzyme activities, and affect pollutant degradation.	Ma et al. (2017)
CeO₂ NPs	Trickling filter/packed-bed biofilm reactor (fixed-bed) — long-term exposure	Long-term exposure alters biofilm thickness, EPS composition and community structure, thereby affecting organic matter and N/P removal performance (clear cumulative effects).	Amirbandeh et al. (2024)
CeO₂ NPs	Activated sludge systems (long-term exposure)	Denitrification deteriorates; key enzyme activities decline; bacterial diversity decreases and network complexity is reduced.	Wang et al. (2018)
Graphene oxide (GO)/carbon-based nanomaterials	Bioelectrochemical reactors (BER/MFC) and aerobic/anaerobic granular sludge systems	GO/carbon nanomaterials are often used for electrode modification to enhance electron transfer and can improve bioelectrochemical performance; however, unmodified or highly accumulated carbon nanomaterials may induce oxidative stress or alter EPS, destabilizing biofilms (bidirectional effects).	Zubair et al. (2024)
CeO₂, ZnO, TiO₂ NPs	Mechanistic synthesis/discussion	Affect microbial membranes and systems via ROS generation, metal ion release, and physical membrane adsorption.	Yang et al. (2013)

#### 2.2.2 Ion release

Metallic nanoparticles (e.g., Ag, ZnO) are capable of generating metal ions in aqueous systems, and these ions pose substantial toxic threats by suppressing protein biosynthesis and altering fundamental metabolic pathways. A well-recognized mechanism of Ag nanoparticle toxicity involves the release of Ag^+^ ions, which interact with intracellular thiol moieties, thereby deactivating key enzymes and disturbing cellular metabolic integrity (Dakal et al., 2016; Vasiliev et al., 2023; Jahan et al., 2024). Similarly, the release of Zn^2+^ ions can induce metabolic disturbances in microbial cells, ultimately inhibiting their growth and reproduction. Several studies have demonstrated a dose-dependent inhibitory effect of ZnO nanoparticles in anaerobic digestion systems. When the concentration of ZnO NPs reaches tens to hundreds of mg g^−1^ VSS, methane production is significantly reduced (e.g., by ~18% and ~75% at 30 and 150 mg g^−1^ VSS, respectively). This reduction is consistent with decreased activities of key methanogenic enzymes (e.g., coenzyme F₄₂₀, acetate kinase) and reduced relative abundance of methanogenic archaea (Wang et al., 2021). Further mechanistic investigations suggest that ZnO toxicity mainly arises from its partial dissolution into Zn^2+^. Moreover, adsorbents such as activated charcoal can mitigate the inhibition of methanogens by sequestering the released Zn^2+^, supporting the conclusion that Zn^2+^ is the primary toxic species (Mu et al., 2023).

#### 2.2.3 Surface effects

Owing to their high surface-to-volume ratios and reactive surface chemistries, nanomaterials tend to associate with diverse environmental biomolecules—proteins, extracellular polysaccharides, and membrane lipids among them—thereby giving rise to protein coronas or other biomolecule-adsorbed interfaces (Rampado et al., 2020). Upon adsorption, these biomolecules often undergo conformational rearrangements or masking of active sites, thereby altering their native structures or blocking their biological functions, such as enzyme activity or ligand recognition (Abarca-Cabrera et al., 2021; Bilardo et al., 2022). These mechanistic pathways have been repeatedly substantiated through *in vitro* molecular mimicry experiments and further corroborated by studies conducted within cellular and environmental settings. For example, nanoparticles can adsorb extracellular polymeric substances (EPS) through electrostatic interactions, weakening the protective barrier of microorganisms (Gao et al., 2025). EPS, an important protective layer surrounding microbial cells, helps microorganisms resist external environmental stresses (Sun et al., 2023). Moreover, adsorption of nanomaterials can disrupt the structure of EPS, thereby weakening microbial defenses (Li et al., 2022). In addition, engineered nanomaterials (including metals, metal oxides, and SiO₂) can directly interact with cell membranes through surface charge, hydrophobicity, and functional groups, altering membrane lipid composition and fluidity. Such interactions may impair the activity of membrane-associated enzymes and transporters, ultimately perturbing cellular metabolic processes (Ahmed et al., 2020; Wei et al., 2022; Modi et al., 2023; Jiang et al., 2024).

#### 2.2.4 Bioaccumulative effects

Certain nanomaterials can accumulate inside cells via microbial metabolic pathways or through intracellular translocation, thereby interfering with normal physiological functions. For example, silver nanoparticles (AgNPs) can attach to and penetrate microbial cells (in eukaryotic microorganisms, this may occur through endocytosis or intracellular transport), where they accumulate and interact with the respiratory chain and enzymatic systems, ultimately inducing oxidative stress and metabolic disorders (Babele et al., 2019; Rasmussen et al., 2022; More et al., 2023). By comparison, the bioaccumulative behavior of nanomaterials is markedly affected by factors such as particle dimensions, surface coatings or modifications, and exposure concentration. For instance, gold nanoparticles of intermediate size (~50 nm) exhibited significantly higher cellular uptake efficiency compared with smaller (20 nm) or larger (74 nm) particles, highlighting the critical role of particle size in regulating intracellular accumulation (Sabourian et al., 2020). Moreover, functional modifications of nanoparticle surfaces (e.g., the introduction of charged functional groups or targeted ligands) can alter their interactions with cell membranes and facilitate cellular entry via receptor-mediated translocation (Sanità et al., 2020). In addition, intracellular accumulation of nanomaterials can trigger oxidative stress, ultimately leading to cellular damage.

#### 2.2.5 Indirect ecological effects

Aside from the mechanisms of cellular disruption previously described (Figure 1), nanomaterials are capable of forming synergistic or antagonistic interactions with various coexisting contaminants—for example, heavy metals or organic compounds—which can substantially alter and complicate their overall behavior and effects within biological treatment systems. A range of empirical studies has shown that the concurrent presence of engineered nanoparticles (ENPs) and heavy metals can facilitate the partitioning of metals into biofilms or sedimentary environments, resulting in an escalation of their toxic potency. For example, co-exposure of TiO₂ nanoparticles with Cd^2+^ significantly increased Cd accumulation and toxicity in biofilms, resulting in reduced metabolic activity (Wang W. et al., 2022). Similarly, silver species (ionic or nano) can inhibit microbial metabolism on their own and exert synergistic inhibitory effects when combined with other chemical stressors (Ipe et al., 2020; Herruzo-Ruiz et al., 2024). In addition, TiO₂ nanoparticles can adsorb and concentrate aqueous-phase organic pollutants (e.g., PAHs, dyes, and antibiotics) on their surfaces, thereby forming a pre-concentrated contaminant–TiO₂ complex. This surface enrichment not only serves as a precursor to TiO₂-mediated photocatalytic degradation but also alters the transport, transformation, and bioavailability of pollutants in the aqueous phase. Consequently, in the presence of microorganisms (e.g., in activated sludge systems), TiO₂–organic matter complexes may change contaminant exposure patterns (or enhance the intracellular availability of certain inhibitory substances), ultimately affecting microbial degradation capacity and overall treatment performance (Xu H. et al., 2021; St Mary et al., 2024).

**Figure 1:**
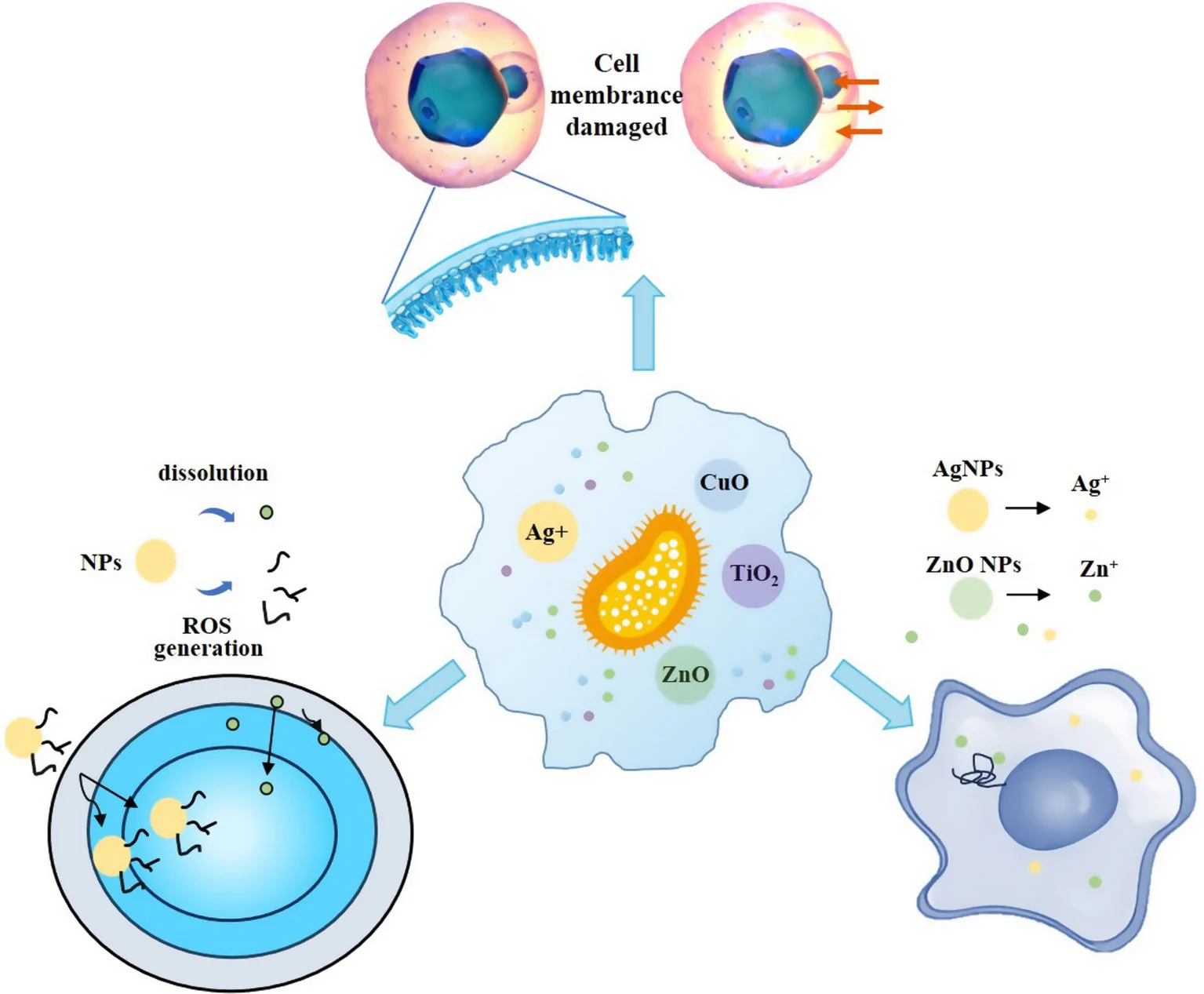
Mechanisms of nanoparticle-induced cytotoxicity via ion release, ROS generation, and membrane damage.

### 2.3 Recovery strategies

#### 2.3.1 Physical and chemical recovery

When the function of a biological treatment system is impaired due to nanomaterial toxicity, nanoparticle concentrations can be reduced and their adverse effects mitigated through various physicochemical recovery. For high-concentration wastewaters, dilution/diversion or controlled release can be applied to lower the instantaneous nanoparticle concentration entering the biological unit, thereby alleviating toxicity pressure on the microbial community. For low- and medium-concentration residuals, high-efficiency adsorbents such as activated carbon and zeolites can be employed, offering an efficient and low-cost option for nanoparticle removal by combining physical adsorption with chemisorption (McGillicuddy et al., 2018; Ramirez Arenas et al., 2023). In addition, chemical oxidation or reduction can further transform residual nanomaterials into low- or non-toxic forms. For example, advanced oxidation approaches like Fenton chemistry and ozonation yield reactive oxygen species (ROS), which subsequently convert nanoparticles into smaller molecular species (He et al., 2014). Alternatively, reducing agents such as sodium sulfide can precipitate metal nanoparticles, significantly lowering their biotoxicity (Fennell et al., 2017; Khodaparast et al., 2022).

#### 2.3.2 Biofortification

When nanomaterials impair the functioning of biological treatment systems, a combined strategy of bioaugmentation and nutrient supplementation can be employed to restore system activity. First, naturally tolerant or degrading strains can be screened or enriched. For instance, *Bacillus* (which secretes extracellular polymers) and *Pseudomonas* (a known metal adsorbent) produce EPSs that can complex or adsorb nanoparticles and released metal ions, thereby altering particle aggregation and dissolution characteristics and reducing toxicity (Naz et al., 2016; Fu et al., 2021; Chen et al., 2022; Mu et al., 2023). Secondly, metal-binding proteins (e.g., metallothioneins) can be introduced into microbial strains through genetic engineering, enabling improved resistance to metals and facilitating the biomobilization or biotransformation of metal contaminants (Yuan et al., 2019). Finally, supplementation of systems such as anaerobic digestion with readily available carbon sources (e.g., acetic acid or sodium acetate) or with adsorbent materials (e.g., activated charcoal) can synergistically restore biochemical functionality and pollutant removal performance. This is achieved both by directly supplying methanogens with substrates to boost metabolic activity and by mitigating toxicity through sequestration of soluble metal ions (Lemaigre et al., 2023).

#### 2.3.3 Biofilm protection

The addition of protective substances (e.g., mineral or colloidal particles, surfactants) to biofilm or effluent systems can mitigate nanomaterial-induced damage to biofilms. On the one hand, minerals or colloids (e.g., kaolin) can heteroaggregate or adsorb with nanoparticles, thereby sequestering them or altering their surface properties and effective concentrations, which reduces direct contact with microbial cells or EPS. On the other hand, surfactants (e.g., polysorbate-80/Tween-80 or biosurfactants/pulmonary surfactants) can form a protective layer at the particle–biotic interface or modify interfacial wetting and charge, thereby significantly reducing nanoparticle uptake by microbial surfaces and their adhesion to biofilms (Ueda et al., 2019; Dong and Zhou, 2020; Radiom et al., 2020).

#### 2.3.4 Nanomaterial substitution

Replacing nanomaterials with greater environmental toxicity at the pollution source, or designing more environmentally friendly alternatives (e.g., capped nanoparticles), represents an important mitigation strategy. For example, capped nanoparticles can reduce direct contact with microorganisms and thereby lower toxicity by coating the nanomaterial surface with a protective layer (e.g., silica or polymer layers) (Sanchez et al., 2017; Wang et al., 2019; Adamska et al., 2021). In addition, the development of biodegradable nanomaterials constitutes another key approach to minimizing environmental risks.

## 3 Impacts, mechanisms and recovery strategies of microplastics on biological treatment systems

The impacts, underlying mechanisms, and recovery strategies related to microplastics in biological treatment systems have become major research hotspots in environmental science and wastewater treatment. Microplastics (MPs), defined as plastic particles smaller than 5 mm, are widely distributed in the natural environment. Owing to their stability, adsorption properties, and potential toxicity, their influence on wastewater treatment processes has attracted growing research interest.

### 3.1 Impact of microplastics on biological treatment systems and their mechanisms

#### 3.1.1 Physical interference

Microplastics systematically affect sludge settling characteristics and microbial community stability through multilevel physical interference mechanisms, including particle adsorption, structural disruption, and hindrance of material transfer, thereby significantly impairing the overall performance of biological treatment systems. Within reactors, they readily bind to sludge particles, altering flocculation properties, increasing sludge viscosity, and reducing settling performance, which ultimately influences hydraulic retention time and treatment efficiency (Maliwan et al., 2021). At environmentally relevant low concentrations, microplastics activate the release of soluble microbial products (SMP) and extracellular polymeric substances (EPS), contribute to the growth of larger sludge floc structures, and negatively affect microbial communities by lowering both their abundance and diversity (Wang Q. et al., 2022). In biofilm systems, microplastics alter surface properties and reduce attachment points, while friction-induced detachment further compromises biofilm stability and pollutant removal capacity (Maliwan et al., 2021). When microplastics are incorporated into activated sludge flocs, they interfere with the original floc structure, reduce the relative abundance of nitrifying and denitrifying microorganisms, and elevate the sludge volume index (SVI), leading to poorer settling characteristics and diminished dewatering performance (Wei et al., 2023). The physical barrier formed by microplastics also restricts substrate and nutrient transport, inhibiting material exchange among microorganisms and consequently lowering the purification efficiency of the system. Notably, particle size and morphology are critical determinants of the interference effect, as smaller particles are more likely to penetrate deeper into sludge flocs, causing more severe damage to microbial communities and system function (Wei et al., 2023).

#### 3.1.2 Chemical effects

Microplastics contribute to increased toxicity in biological treatment systems by co-adsorbing pollutants and releasing chemical additives, processes that perturb microbial activity, functional stability, and community organization, ultimately undermining treatment performance and ecological safety (Figure 2). First, the combination of extensive surface area and hydrophobic surface properties allows microplastics to efficiently sorb hydrophobic persistent organic pollutants (e.g., PAHs, PCBs) together with inorganic metal ions in aquatic environments, enabling them to serve as effective pollutant carriers that markedly influence contaminant transport dynamics and environmental longevity (Gao et al., 2021; Wang L. C. et al., 2024). Pollutants adhering to microplastic surfaces can form complexes that exert compounded toxicity on microbial communities upon entering treatment units, particularly inhibiting nitrifying and denitrifying bacteria and reducing nitrogen removal efficiency (Wang Z. et al., 2020). Moreover, the adsorption of heavy metals onto microplastics is often enhanced by biofilm formation or environmental weathering. These adsorbates can subsequently desorb during treatment (e.g., under shifts in hydraulic or chemical conditions, or during digestion and consumption processes), leading to secondary contaminant release and increased perturbation of microbial communities (Gao et al., 2021; Qi et al., 2021; Emecheta et al., 2024). In addition, microplastic–pollutant complexes may exacerbate ecological risks through particle transport and trophic transfer.

**Figure 2:**
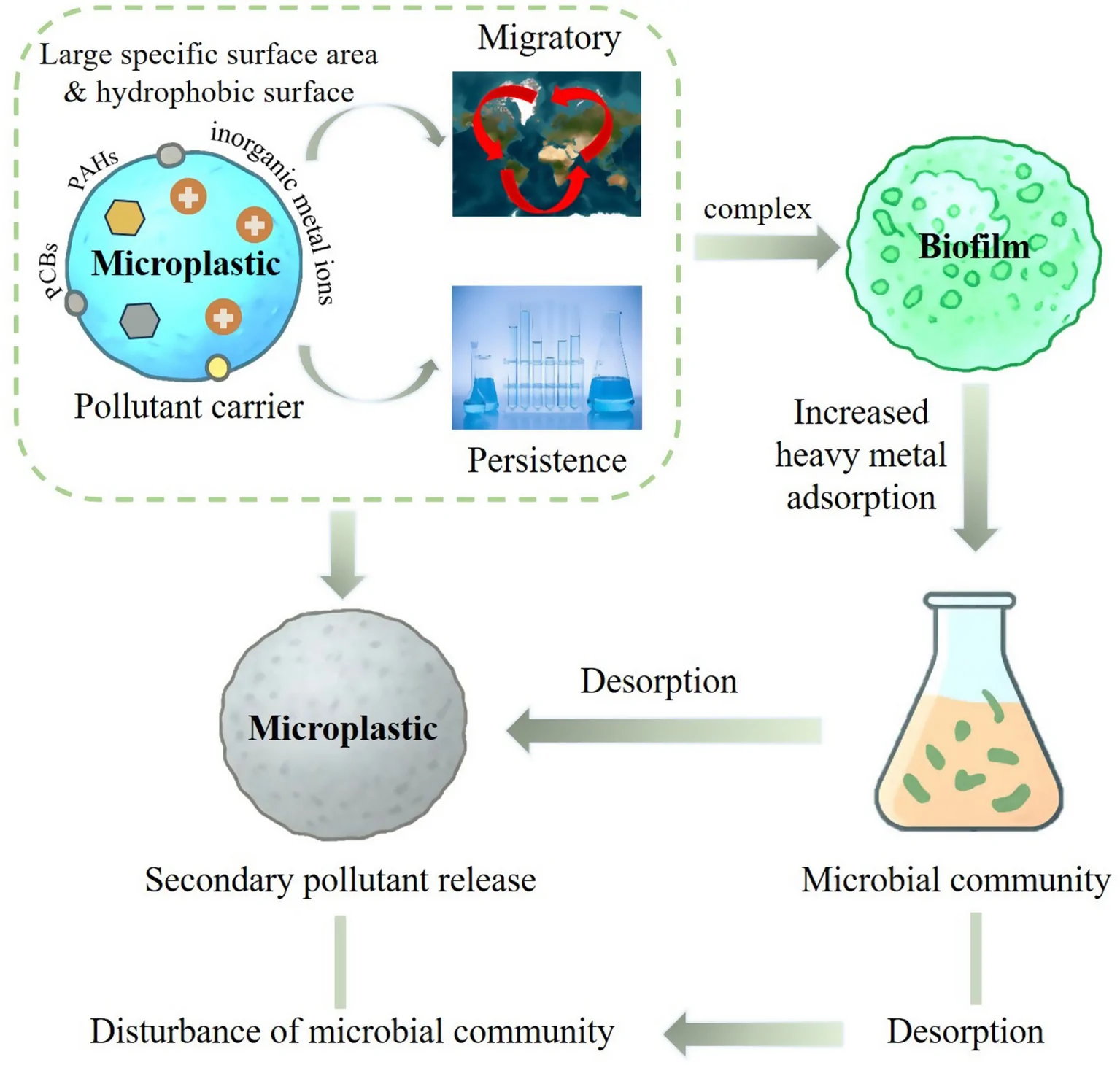
Schematic illustration of the interactions between microplastics, biofilms, and microbial communities.

Second, chemical additives [e.g., bisphenol A (BPA), phthalates (PAEs)] embedded in microplastics are gradually released into wastewater and sludge systems. The presence of such additives imposes metabolic stress on microbial communities, leading to enzymatic inhibition and transcriptional alterations that collectively compromise organic matter degradation and nitrogen cycling (Cydzik-Kwiatkowska et al., 2021; Ferrer-Polonio et al., 2021; Shao et al., 2022). Furthermore, PAEs (e.g., DBP, DEHP) significantly alter microbial community composition and reduce pollutant removal efficiency in activated sludge or mixed system (Wang Q. et al., 2020). The co-occurrence of microplastics with additives or other environmental contaminants has been shown to amplify inhibitory pressures on nitrification processes and select for tolerance and resistance genes, thereby elevating the potential for microbial community dysfunction (Wang Q. et al., 2020; Wang Z. et al., 2020; Yan et al., 2022).

#### 3.1.3 Biological toxicity

Microplastics impair the physiological function and ecological stability of microbial communities through membrane disruption, oxidative stress, and gene expression regulation, thereby reducing the purification efficiency and long-term stability of biological treatment systems. First, they can induce both physical and chemical damage via direct interaction with microbial cell surfaces. Rough or sharp edges of fibrous or fragmented microplastics may damage the cell wall and membrane through friction or puncture at high concentrations (e.g., twisting of algal thylakoids and membrane rupture have been observed experimentally), leading to the release of intracellular substances and loss of cellular functions (Lu et al., 2022). In addition, adsorption of microplastic particles depletes available membrane surface area and increases membrane tension, causing destabilization and a significant reduction in membrane lifespan—a physical stretching mechanism confirmed in model lipid bilayers and erythrocytes (Fleury and Baulin, 2021). The surface chemistry of microplastics, including hydrophobicity, functional groups, and charge, further modulates their affinity for membranes and membrane proteins, which can enhance particle–membrane adhesion or exacerbate dysfunction by inducing oxidative stress or interfering with membrane-associated enzymes and channels. Experimental evidence has demonstrated alterations in membrane integrity in cellular models subjected to mitochondrial and oxidative stress, resulting in reduced microbial activity and community dysfunction (Saenen et al., 2023).

Exposure to microplastics and their weathered derivatives elevates intracellular ROS levels in microorganisms, consequently triggering oxidative stress. This is typically manifested by upregulation of antioxidant enzymes, elevated lipid peroxidation, and consequent damage to membranes, proteins, and DNA, which inhibit microbial metabolism and growth (Jasińska et al., 2022; Gao et al., 2023; Xiang et al., 2023). Moreover, microplastic exposure reorganizes gene expression in microbial communities. On the one hand, stress and antioxidant pathways are activated (e.g., elevated ROS and concomitant antioxidant responses), indicating a protective strategy to improve tolerance (Jasińska et al., 2022). On the other hand, transcriptome analyses reveal significant perturbations in genes associated with protein synthesis, ribosomal biogenesis, energy metabolism, and growth (often downregulated), while genes linked to carbon and alkane degradation, as well as related transporters (e.g., fadL, alkM), are selectively upregulated. This indicates that microplastics reconfigure microbial metabolic pathways and alter material fluxes (Gao et al., 2023; MacLean et al., 2024). Such remodeling of gene expression patterns is accompanied by changes in the abundance and activity of functional genes (e.g., nitrification/denitrification genes), ultimately disrupting community metabolic networks and treatment processes, and reducing nitrogen and organic matter removal efficiency (Seeley et al., 2020).

### 3.2 Recovery strategies

#### 3.2.1 Physical pre-treatment

The mitigation of microplastic-induced stress in biological treatment systems often relies on physical recovery approaches, which are valued for their straightforward application and rapid remedial outcomes. First, installing pretreatment units (e.g., screening, flotation, and sedimentation) before wastewater enters the biological treatment system, the microplastic load entering the system can be significantly reduce (Iyare et al., 2020). Microplastic removal performance exhibits a strong dependence on particle size, with larger particles being efficiently removed by screening and sedimentation, while finer particles require advanced separation techniques and are typically removed less effectively (Bilgin et al., 2020; Carnevale Miino et al., 2024). In addition, sedimentation tanks have been shown to be effective in removing denser microplastic particles (e.g., PVC and PET) by gravity, further reducing the burden on the subsequent biological treatment system; they have performed well for particles of various shapes and sizes under steady-state settling conditions, but remain challenging for filamentous and very small (<1 mm) particles (Liu et al., 2021; Xu Z. et al., 2021). Second, for microplastics that have entered the reactor, sedimentation and filtration techniques can achieve efficient separation and removal. For example, the inclined plate settling technology significantly improves the separation efficiency of microplastic particles by increasing the settling area, which is suitable for rapid treatment under high load conditions (Tarpagkou and Pantokratoras, 2014). Precision filtration systems (e.g., ultrafiltration, microfiltration) can effectively retain smaller microplastic particles owing to their fine pore sizes (Sharma et al., 2023), but it is important to note that these systems have high operating costs and are susceptible to membrane contamination and clogging, and therefore need to be optimized in conjunction with pretreatment measures. In recent years, multilayer media filters developed based on the principle of depth filtration have shown higher removal efficiencies with lower energy consumption, and have become one of the development directions of physical treatment technologies (Zhang et al., 2023).

#### 3.2.2 Chemical recovery

In microplastic-induced disturbances of bioprocessing systems, chemically modulated recovery show considerable promise as effective adjuncts. First, chemical modification of microplastic surfaces can enhance their hydrophilicity or promote aggregation and settlement, thereby mitigating negative impacts on the system (Bule Možar et al., 2023). For example, oxidative modification with oxidizing agents (e.g., persulfates) or photocatalytic techniques can introduce hydrophilic functional groups, thereby enhancing microbial attachment and microplastic biodegradation. In addition, chemical cross-linking agents or composite flocculants (e.g., natural polymers such as chitosan combined with traditional inorganic/polymer coagulants) can induce the formation of larger particles or flocs, thereby significantly improving settling performance and facilitating subsequent physical separation or membrane filtration (Huang et al., 2023). The removal of small- and medium-sized microplastics can be significantly improved by the application of optimized flocculation–coagulation combinations (Lapointe et al., 2020). However, excessive chemical use should be avoided to prevent inhibitory effects on system microorganisms.

Secondly, the addition of adsorbents in the reactor represents another important means of chemical modulation. Activated carbon provides strong adsorption capacity for microplastics because of its extensive surface area and pore-rich structure, making it especially effective for removing nano- and small-sized fractions (Anuwa-Amarh et al., 2024). Zeolites, in contrast, utilize surface adsorption and ion-exchange capacities to effectively remove microplastics and their associated harmful contaminants (Munir et al., 2024; Omorogie and Helmreich, 2024). In recent years, bio-based adsorbents such as chitosan and cellulose derivatives have gained growing interest because of their environmental compatibility, renewable nature, and strong adsorption performance, underscoring their sustainability and ecological advantages (Ullah et al., 2025).

#### 3.2.3 Biofortification

In response to the functional degradation of biological treatment systems caused by microplastic pollution, biofortification strategies have gradually attracted increasing research and application interest due to their green, safe, and sustainable characteristics (Cai et al., 2023). First, the introduction of tolerant or degradation-capable bacterial consortia can effectively improve the system’s adaptability and degradation potential toward microplastics. The bacterial strain *Pseudomonas aeruginosa* AKS2 has been reported to achieve nearly 5% LDPE degradation within 45 days under non–pre-oxidized conditions, exhibiting a markedly higher efficiency compared with prior studies (Tribedi and Sil, 2013). Strong surface adhesion and degradation potential have also been observed among fungi across several phyla, notably Ascomycota, Basidiomycota, and Mucoromycota, which include classes such as Dothideomycetes, Agaricomycetes, and Mucoromycetes (Ekanayaka et al., 2022). Therefore, targeted screening, enrichment, and dosing of these strains not only enhance system tolerance to microplastics but also accelerate biodegradation and improve overall system stability.

Notably, the application of genetically engineered bacteria offers new opportunities for efficient microplastic degradation. Genetic engineering enables the heterologous expression of plastic-degrading enzymes, such as PETase and MHETase, in widely used host organisms such as *Escherichia coli* (*E. coli*), thereby generating functional strains with enhanced capability for polyethylene terephthalate (PET) degradation (Wang T. et al., 2024; Jiang et al., 2025). With advances in precision gene-editing technologies such as PlastiCRISPR, strain tolerance, enzyme expression efficiency, and substrate specificity can be further optimized, making microplastic degradation more targeted and efficient. However, the ecological safety of these engineered bacteria must be comprehensively evaluated during application to prevent secondary environmental risks (Palit et al., 2025).

Additionally, stimulating the degradation potential of microorganisms through co-metabolism strategies has proven effective. During co-metabolism, microorganisms degrade easily utilizable substrates (e.g., glucose, methanol, ethanol) while simultaneously decomposing recalcitrant microplastics. Recent studies have shown that microbial communities from plastic-contaminated environments can utilize plastics as carbon sources. For example, ConsPlastic-A and ConsPlastic-B complexes established from landfill soils utilized polyethylene and PET as carbon sources and revealed numerous novel plastic-degrading microorganisms and associated enzymes through metagenomic analysis (Roman et al., 2024). This strategy not only enhanced microplastic removal but also increased microbial activity and community functional diversity, thereby contributing to rapid recovery and long-term system stability (Gao et al., 2024).

#### 3.2.4 Alternative materials

Among the diversified microplastic management strategies, source control is gradually gaining attention, and the promotion of alternative materials is considered one of the most forward-looking and sustainable approaches. First, promoting the use of biodegradable plastics [e.g., polylactic acid (PLA), polyhydroxyalkanoates (PHA)] can effectively reduce the input of conventional plastics at source and lower the risk of subsequent microplastic generation (Mukherjee and Koller, 2023). Biodegradable plastics can be decomposed into water, carbon dioxide, and biomass by microorganisms in the natural environment, thereby avoiding the long-term accumulation of plastics and the formation of microplastics (Fayyazbakhsh et al., 2025). Studies have shown that materials such as PLA exhibit good degradability in wastewater treatment systems and are significantly less toxic to microbial communities than traditional petroleum-based plastics, further reducing the potential threat to treatment system stability (Nik Mut et al., 2024). In addition, promoting such materials not only helps mitigate microplastic pollution, but also reduces dependence on petroleum resources, fosters the development of a circular economy, and achieves both environmental and economic benefits.

On the other hand, replacing toxic chemical additives used in conventional plastics with green additives is also a key strategy for reducing the ecological risks associated with microplastics. Conventional plastic additives, including phthalates and bisphenol A, have been widely reported to suppress microbial growth and metabolic activity, thereby impairing the performance of biological treatment systems (Dueñas-Moreno et al., 2022; Rybczyńska-Tkaczyk et al., 2023). In contrast, novel plant-based plasticizers (e.g., citrate, epoxidized soybean oil) and environmentally friendly stabilizers not only preserve the physical properties of plastics but also significantly reduce toxicity to microorganisms and disturbance of community structure (Xu et al., 2019; Intranuwong et al., 2025). More importantly, the use of green additives can decrease the environmental persistence and bioaccumulation of microplastics, thereby fundamentally mitigating the risk of their spread in aquatic environments and food chains (Ali et al., 2023). Through the synthesis of current evidence, the mechanisms by which microplastics affect biological treatment systems are better clarified, and the role of scientifically designed restoration strategies in improving system efficiency and sustainability is emphasized.

## 4 Impacts, mechanisms and recovery strategies of PFAS on biological treatment systems

Per- and polyfluoroalkyl substances (PFAS) are chemically stable synthetic compounds widely employed in waterproofing, antifouling, and heat-resistant products. Their environmental persistence, limited degradability, and potential toxicity have positioned PFAS as critical contaminants in global water pollution, prompting growing research interest in their behavior and removal within biological treatment systems.

### 4.1 Impacts and mechanisms of PFAS on biological treatment systems

#### 4.1.1 Microbial community impacts

In biological treatment systems, the microbial community serves as the core functional unit for pollutant degradation and nutrient cycling, and its activity and composition directly determine the stability and treatment efficiency of the system. Persistent organic pollutants entering treatment systems over time, especially PFAS, create sustained toxic stress that threatens the stability of microbial communities. On the one hand, PFAS can inhibit microbial growth and metabolic activity, showing especially strong inhibitory effects on key functional groups (e.g., nitrifying and denitrifying bacteria) (Lu et al., 2022; Tang et al., 2022). This inhibition is mainly attributed to PFAS-induced disruption of microbial cell membranes, which compromises membrane integrity and selective permeability by disturbing the lipid bilayer structure. As a result, cellular osmotic balance and energy metabolism are impaired, ultimately leading to decreased physiological activity or cell death (Cao et al., 2022a). Mechanistically, PFAS molecules generally consist of a long-chain fluorocarbon backbone and hydrophilic groups (e.g., carboxyl or sulfonic acid), conferring strong amphiphilic properties. This coexistence of hydrophobic and hydrophilic characteristics endows PFAS with high surface activity in aqueous environments, enabling both strong binding to aqueous contaminants and strong interactions with lipid bilayers of microbial membranes (Naumann et al., 2022; Musegades et al., 2024). Specifically, model membrane and microbial experiments have demonstrated that the hydrophobic fluorocarbon chains of PFAS can insert into phospholipid mono−/bilayers, perturb lipid packing, and increase membrane permeability (Cao et al., 2022a). In bacterial and cellular systems, such membrane damage is often accompanied by elevated ROS production, impaired membrane resistance, and disrupted osmotic regulation, which together disturb energy metabolism and signaling and ultimately inhibit physiological activity (Yin et al., 2023) (Figure 3). Furthermore, the chain length of PFAS strongly influences their environmental behavior and microbial toxicity. Long-chain PFAS (e.g., PFOA, PFOS), owing to their larger molecular weight and higher hydrophobicity, tend to bind more readily to organic matter in water or microbial surfaces, forming stable complexes that are difficult to degrade (Liu and Li, 2024). Chain length also determines environmental partitioning and biological effects: long-chain PFAS are more readily adsorbed by sludge, biofilms, and organisms, leading to stronger toxicity and greater bioaccumulation, thereby exacerbating the inhibition of microbial functions in biological treatment systems (Fitzgerald et al., 2018; Pickard et al., 2022). In contrast, short-chain PFAS show lower adsorption affinity, greater mobility in aqueous phases, and lower acute toxicity in various assays (Riegel et al., 2023). Nevertheless, a few reports have documented partial microbial transformation or defluorination of some PFAS under specific microbial or extreme reaction conditions (observed under strict anaerobic/enrichment or specific strains), suggesting the possibility that “short chains may be partially metabolized by microorganisms under certain conditions”, but that such transformations are usually limited and condition-dependent (Huang and Jaffé, 2019).

**Figure 3:**
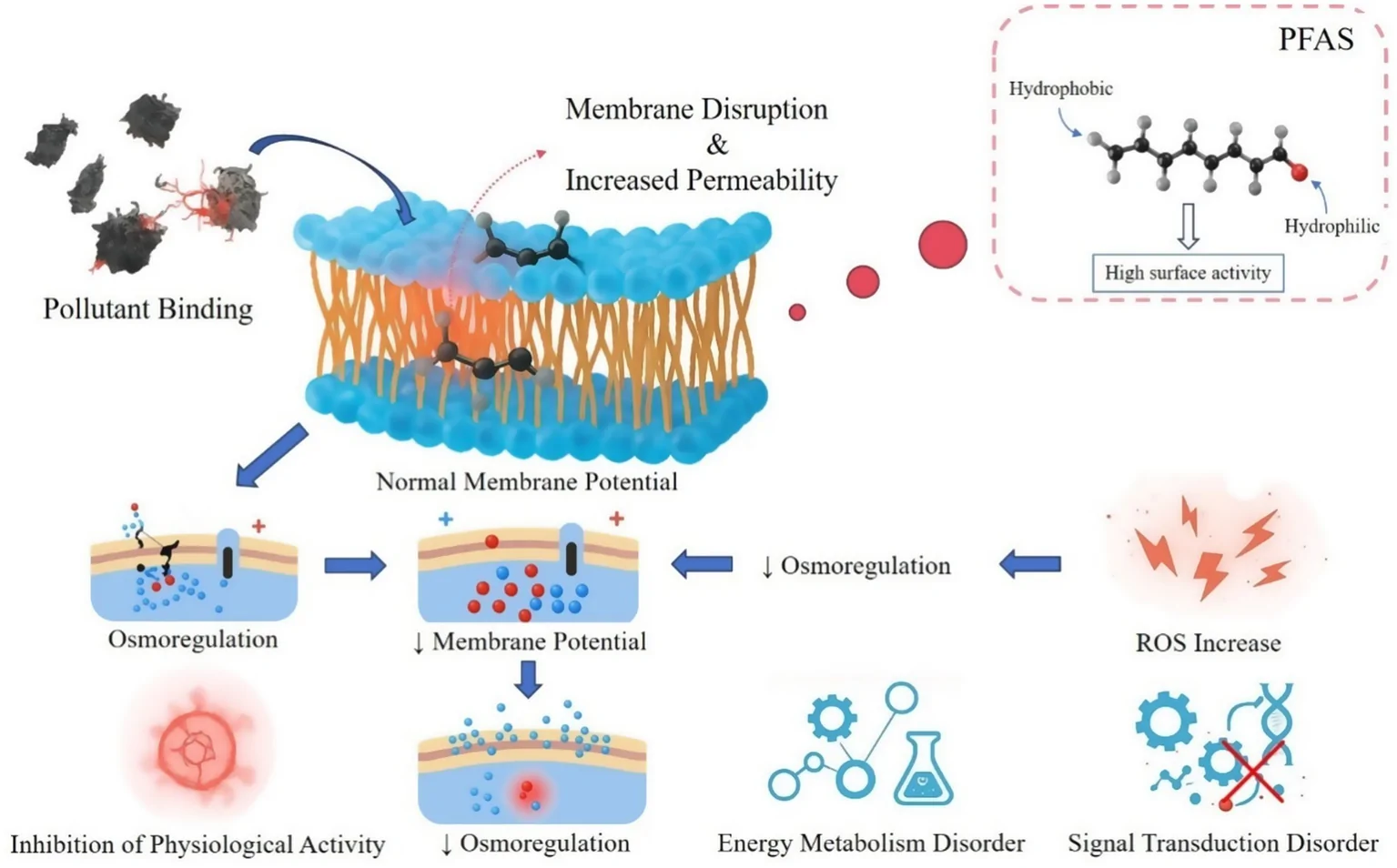
Schematic illustration of PFAS-induced cell membrane disruption and physiological dysfunction mechanisms.

In addition, long-term accumulation of PFAS can induce overproduction of intracellular reactive oxygen species (ROS) in microbial cells, thereby triggering oxidative stress (Liu et al., 2016). The accumulation of PFAS can disrupt cellular redox homeostasis by elevating intracellular ROS levels, thereby causing oxidative damage to lipids, proteins, and genetic material (Qi et al., 2020). Moreover, PFAS interfere with microbial metabolism and stress responses by modulating gene expression (Huang et al., 2022b; Tang et al., 2022). PFAS have been shown to influence transcriptional regulation, thereby modifying the expression of genes governing degradative metabolism, antioxidant capacity, and lipid metabolism (Wen et al., 2021; Hofmann et al., 2023).

On the other hand, sustained PFAS exposure also results in profound alterations of microbial community structure, typically manifested as reduced community diversity and shifts in dominant populations. Under PFAS stress, tolerant strains may proliferate and outcompete sensitive taxa that play crucial roles in pollutant removal, ultimately driving a decline in overall system performance (Shittu et al., 2023).

#### 4.1.2 Biological treatment process impacts

Furthermore, PFAS are not only toxic to microorganisms themselves but also significantly impair the overall efficiency of biological processes through multiple mechanisms. Specifically, PFAS indirectly affect microbial carbon, nitrogen, and phosphorus cycling by altering microbial community composition (Lin et al., 2025). By interfering with microbial metabolic pathways, PFAS inhibit nitrification and denitrification processes, ultimately leading to a significant decrease in nitrogen removal efficiency (Lu et al., 2022). PFAS exposure has also been shown to negatively impact biological phosphorus removal by inhibiting the activity of polyphosphate-accumulating organisms (Qian et al., 2024). In addition, the persistence of PFAS may disrupt the degradation of organic matter. Under PFAS stress, some microorganisms preferentially activate stress response mechanisms rather than degrade organic substrates; meanwhile, the adverse effects of PFAS on extracellular enzyme activities and electron transfer processes further limit the effective degradation of organic matter (Cao et al., 2022a). Excessive PFAS loading can markedly suppress organic matter biodegradation and induce the accumulation of residual organic pollutants, posing a threat to the stability and long-term performance of treatment systems (Cao et al., 2022b).

#### 4.1.3 Sludge disposal issues

In addition to the direct impacts mentioned above, the accumulation of PFAS further increases the complexity of sludge disposal. Because of their stability and hydrophobicity, PFAS readily accumulate in sludge over time, increasing sludge toxicity and restricting its safe disposal and resource-oriented utilization (e.g., agricultural land application or land reclamation) (Oza et al., 2025). The accumulation of PFAS in sludge—particularly at high concentrations of PFOA—can increase the total content and protein fraction of extracellular polymeric substances (EPS), and alter the secondary structure and floc morphology of EPS. These changes result in sludge floc loosening and deterioration of dewatering indices such as specific resistance to filtratio and capillary suction time (Xie et al., 2022; Li et al., 2023). Experimental evidence further indicates that an increase in soluble EPS alters the rheological properties of sludge (e.g., viscosity, yield stress), which directly leads to reduced dewatering performance and higher dewatering costs (Liang et al., 2020; Li et al., 2023). This not only increases the difficulty of subsequent sludge transportation, disposal, and resource utilization, but also imposes higher demands on the economic feasibility and sustainable operation of wastewater treatment plants.

#### 4.1.4 Synergies between PFAS and other pollutants

PFAS may act synergistically with other contaminants in water (e.g., heavy metals, pharmaceutical residues), thereby exacerbating the burden on biological treatment system (Cai et al., 2019; Li et al., 2020). PFAS may also contribute to increased microbial toxicity by transporting co-contaminants and improving their bioavailability (Huang et al., 2022a).

### 4.2 Recovery strategies

#### 4.2.1 Physicochemical recovery

In response to the persistence and recalcitrance of PFAS in biological treatment systems, physicochemical remediation strategies have recently been recognized as effective means to mitigate their negative impacts and enhance system stability. Among these, adsorption and separation technologies are widely applied due to their operational simplicity and adaptability. Specifically, the introduction of efficient adsorbents (e.g., activated carbon, zeolite, and iron oxide nanoparticles) at the front end of the system or during the treatment process can effectively reduce PFAS concentrations in influent water and alleviate the toxicity burden on both microbial communities and treatment processes (Omo-Okoro et al., 2021; Lee et al., 2023; Ponge et al., 2024). Activated carbon exhibits excellent adsorption performance for long-chain PFAS, including PFOA and PFOS, enabling efficient removal (Ponge et al., 2024), whereas iron oxide nanoparticles, benefiting from their large surface area and surface activity, can similarly capture PFAS and enable their removal via solid–liquid separation (Lee et al., 2023).

Advanced oxidation processes are regarded as promising chemical strategies for PFAS removal due to their ability to attack C–F bonds and promote the gradual mineralization of PFAS into harmless byproducts (Zango et al., 2023). AOPs such as ozonation, photocatalysis, and Fenton-based methods generate highly oxidizing hydroxyl radicals that can effectively break PFAS molecular chains (Huang et al., 2016; Leonello et al., 2021). Photocatalytic oxidation has been reported to be more advantageous for the degradation of short-chain PFAS (Olatunde et al., 2020), whereas Fenton’s reagent exhibits higher efficiency in treating long-chain PFAS. Importantly, this technology not only significantly reduces PFAS concentrations but also synergizes with biological treatment processes, thereby further improving overall pollutant removal (Santos et al., 2016).

#### 4.2.2 Biofortification

In response to the high persistence and recalcitrance of PFAS in biological treatment systems, bio-enhanced remediation strategies have recently emerged as effective approaches to improve system resilience and purification performance (Douna and Yousefi, 2023). Firstly, screening for and introducing microorganisms with high tolerance or potential degradation capacity toward PFAS has been demonstrated as a practical enhancement measure. Some Pseudomonas spp., for instance, are not only able to tolerate elevated PFAS concentrations but also possess certain degradation potential (Chetverikov et al., 2023; Chiriac et al., 2023). The introduction of such functional strains can improve the overall resistance and stability of the microbial community, thereby alleviating PFAS-induced toxic stress and enhancing the sustained operation of the system.

Meanwhile, with advances in molecular biology and genetic engineering, the use of gene-editing technologies to construct engineered strains targeting PFAS degradation has received increasing attention (Olawade et al., 2025). By introducing genes encoding specific degradation enzymes (e.g., dehalogenases), the ability of engineered bacteria to cleave the C–F bond and degrade PFAS can be significantly enhanced. Studies have shown that PFAS removal is markedly improved following the successful expression of these enzymes in engineered strains (Harris et al., 2022).

#### 4.2.3 Multi-process coupling

In response to the high persistence and recalcitrance of PFAS in biological treatment systems, multi-process coupling technologies have received extensive attention in recent years. The integration of different treatment units can complement each other’s advantages, synergistically enhance PFAS decomposition and removal efficiency, and provide a basis for restoring system function and stable operation (Jin et al., 2021).

Specifically, the Anaerobic-Aerobic Combined Process is considered a promising treatment strategy. In the anaerobic stage, the carbon-fluorine skeleton of PFAS is disrupted via reductive defluorination to reduce its stability, followed by the aerobic stage, which further oxidizes and degrades intermediate products to achieve more complete mineralization (Niu et al., 2024). This combined process has been reported to not only significantly enhance overall PFAS removal efficiency but also provide favorable conditions for the recovery and functional restoration of the microbial community.

Membrane Bioreactors (MBRs) also show excellent potential for PFAS treatment due to their separation capabilities and enhanced biodegradation functions. The membrane module can effectively retain PFAS and its degradation by-products, reducing their transfer to subsequent treatment stages. Simultaneously, the membrane environment provides stable and concentrated growth conditions for microorganisms, promoting continuous and efficient degradation (Judd, 2008). Long-term studies have demonstrated that the combined process of MBR and granular activated carbon can efficiently (>80%) remove micropollutants, including PFAS, from municipal wastewater, while consuming fewer resources and incurring lower costs than conventional treatment methods (Baresel et al., 2024). In addition, Bioelectrochemical Systems have been recognized as an innovative approach to promote PFAS degradation (Yang and Liu, 2025).

To facilitate cross-contaminant comparison, representative environmental concentrations, study-specific effect concentrations, and treatment impacts of nanomaterials, microplastics, and PFAS in biological wastewater treatment systems are summarized in Table 2.

**Table 2:** Comparative summary of environmental concentrations, study-specific reported effect concentrations, and treatment impacts of nanomaterials, microplastics, and PFAS in biological wastewater treatment systems.

Contaminant	Environmental occurrence in WWTPs	Study-specific experimental exposure or reported effect level	Reported impact on biological treatment	Treatment system/exposure	References
Ag engineered particles	0.11–0.33 μg/L in influent; 1.45–1.65 μg/L in activated sludge; 0.01–0.04 μg/L in effluent	No universal threshold established; field levels were mainly used for occurrence/fate assessment	Decreased soluble COD removal; suppressed biofilm viability; overall removal of Ag engineered particles was 82–95%; >99% of Ag particles discharged in effluent were nanosized	MBBR	Nabi et al. (2021)
TiO2 engineered particles	70–670 μg TiO₂/L in influent; 3,570–6,700 μg/L in activated sludge; 7–30 μg/L in effluent	Study-specific; no universal effect threshold	Overall removal was 90–96%; 55–100% of effluent TiO₂ particles were nanosized	WWTP/biological treatment	Nabi et al. (2021)
AgNPs	Field occurrence is generally in the μg/L or lower range; treatment-specific occurrence differs among plants	Average 10.8 μg/L: no significant inhibition; 131 μg/L for 18 d and 631 μg/L for 5 d: significant inhibition	Reduced soluble COD removal and biofilm viability; approximately **22** and **25%** declines in biofilm viability under the higher exposure conditions	High-rate MBBR	Alizadeh et al. (2019)
ZnO NPs	Environmental occurrence has been reported in wastewater; concentration varies substantially among WWTPs	5 and 10 mg/L: slight inhibition; 20 mg/L: NH₄^+^-N removal decreased from 82.4 to 20% after 5 d; 50 mg/L: decreased from 88.3 to 0%	Strong inhibition of nitrification and altered nitrifying bacterial community at higher exposure levels	Activated-sludge SBR	Wang et al. (2015)
ZnO NPs	—	1 and 5 mg/L: no obvious effect on P removal; 10 mg/L: P removal decreased by ~20%; 20 mg/L: decreased by ~75%	Impaired biological phosphorus removal and altered microbial community diversity at higher exposure	Activated-sludge SBR	Wang et al. (2015)
ZnO NPs (size-dependent)	—	2–60 mg/L, particle sizes 15, 50 and 90 nm; effects became more evident above 10 mg/L	COD and N-removal inhibition was concentration- and particle-size-dependent; smaller particles showed stronger effects in several endpoints; EPS increased markedly at 60 mg/L	SBR	Wang S. et al. (2020)
CuO NPs	—	1 mg/L, long-term exposure	Increased effluent ammonia and TP and inhibited ammonia monooxygenase, nitrite oxidoreductase and phosphorus-related enzymatic activities; bacterial community composition was altered	Activated-sludge system	Wang et al. (2017)
TiO₂ NPs	—	1–50 mg/L; at 50 mg/L significant effects were observed	In aerobic granular sludge at 50 mg/L, COD, NH₃-N and PO₄^3−^-P removal decreased by 20.9%, 12.2%, and 35.1%, respectively	Aerobic granular sludge/algal–bacterial granular sludge, 10 d	Kedves et al. (2024a)
CeO₂ NPs	—	5, 20 and 50 mg/L, 30 d; strongest responses at 50 mg/L	At 50 mg/L, intracellular ROS increased by 38.1% and LDH by 29.0%; phenol, TN and TP removal declined from 98.6 → 96.5%, 53.7 → 49.7%, and 13.0 → 9.0%, respectively	Trickling bioreactor	Amirbandeh et al. (2024)
Microplastics (overall)	177–517 particles/L in influent wastewater from four WWTPs in Chengdu, China	Field occurrence rather than a toxicity threshold	Overall MP removal was 89.3–94.6%; particles <0.2 mm were removed less efficiently (37–45%)	Four municipal WWTPs	Lv et al. (2025)
PP microplastics	—	0.14–0.30 g/L	Inhibited microbial growth and increased SMP/EPS secretion, but did not significantly depress COD or NH₄^+^-N removal; at 2.34–5.00 g/L, changes in microbial diversity and membrane fouling	MBR, long-term accumulation experiment	Wang Q. et al. (2022)
PS microplastics	—	10 and 100 mg/L; 100 mg/L produced clear effects	100 mg/L decreased the specific nitrate reduction rate and caused NO₃^−^-N accumulation; nitrogen-cycling genes including narG, napA, nirS, and nosZ were affected	SBR	Wang et al. (2023)
PE microplastics	—	20 and 200 particles/L as reported by the study	ROS increased by 146.3 and 191.4%, while LDH increased by 185.7 and 316.7%, respectively; denitrification and phosphorus-related enzyme activities were inhibited, whereas AMO activity remained relatively stable	Aerobic granular sludge, 45 d	Zheng et al. (2022)
PVC microplastics	—	0.1 and 0.5 g/L	At 0.1 g/L, nitrite-removal performance decreased by 19.2%; at 0.5 g/L, NH₄^+^-N and NO₂^−^-N removal decreased by 6.2 and 11.6%, respectively	Anammox sludge	Tang et al. (2021)
PBS microplastics	—	0.1 and 0.5 g/L	Effects were less inhibitory than PVC in the anammox system; PBS promoted enrichment of *Ca.* Brocadia under the tested conditions	Anammox sludge	Tang et al. (2021)
PBS microplastics	—	50 and 100 mg/L	COD removal decreased from 95.34% (control) to 89.13 and 86.08%; TP removal decreased from 74.14 to 38.94 and 36.45%	Adsorption–biological coupling system	Liu et al. (2024)
PS microplastics	—	50 and 100 mg/L	COD removal decreased from 96.84% (control) to 88.43 and 87.02%; TP removal decreased from 71.73 to 39.50 and 33.09%	Adsorption–biological coupling system	Liu et al. (2024)
PFAS (sum of detected compounds)	93.2–346.2 ng/L total detected PFAS in influent wastewater; mean PFOA and PFOS concentrations approximately 10.7 and 13.1 ng/L, respectively	Field occurrence; not a toxicity threshold	PFAS were persistent through conventional treatment; activated sludge could transform some longer-chain/precursor compounds and increase short-chain PFCAs	9 municipal WWTPs, USA	Kim et al. (2024)
PFOS	Mean/median concentrations across surveyed WWTPs approximately 13.1/11.0 ng/L	10 and 1,000 μg/L, 48 d	TN removal decreased by 22.48 and 16.30%, respectively; sludge properties, microbial community composition and enzymatic activity were altered	Activated-sludge SBR	Lu et al. (2022)
PFOA	Mean concentration in surveyed U.S. WWTP influents approximately 10.7 ng/L	0.1–1.0 mg/L	At 1 mg/L, anaerobic P release and aerobic P uptake were inhibited by approximately 60 and 50%, respectively; nitrate and nitrite reduction decreased by approximately 13.1 and 5.8%	Aerobic granular sludge	Yang et al. (2020)
PFOA	—	5–50 mg/L	NH₄^+^-N removal decreased from 93.90 ± 3.64% to 77.81 ± 6.86%; NO₂^−^-N removal decreased from 98.6 ± 1.84% to 77.96 ± 1.88%	Anammox system, long-term	Tang et al. (2022)
PFOA	—	0.5 and 20 mg/L	0.5 mg/L inhibited nitrification; at 20 mg/L, TN removal increased from 78.7 ± 6.89% to 86.8 ± 6.39%, illustrating that treatment responses are not necessarily monotonic with concentration	Biological nutrient removal system	Chen et al. (2021)
PFOA	—	0.1–5 mg/L, 84 d	0.1 mg/L showed a slight stimulatory response for ammonia removal, whereas 2 and 5 mg/L produced pronounced inhibition	SBR	Wang H. et al. (2024)
PFOS	—	0.1 and 1 mg/L	TN/TP removal decreased significantly; denitrification rate was inhibited by approximately 25%, while P uptake and P release were inhibited by approximately 73.2 and 73.3%, respectively	Aerobic granular sludge	Wang K. et al. (2024)
PFOS	—	0.1, 0.5 and 5 mg/L	PFOS removal was 89.23 ± 0.92%, 71.92 ± 1.45%, and 48.15 ± 1.90%, respectively, mainly through adsorption; treatment-process responses were concentration- and process-dependent	Aerobic granular sludge	Xu et al. (2023)
PFOA	—	5–20 mg/L	COD removal decreased by approximately 8.85% in the anaerobic unit and 8.31% in the aerobic unit; NH₄^+^-N removal remained relatively stable under the tested conditions	Tandem AnSBR–ASBR	Cao et al. (2022b)

As shown in Table 2, the impacts of emerging contaminants on biological treatment systems vary considerably across contaminant classes and experimental conditions. Nanomaterial-induced effects are generally associated with particle concentration, size, dissolution behavior, and surface properties, whereas microplastic effects are additionally influenced by polymer type, particle size, morphology, aging, and surface-associated contaminants. PFAS responses are affected by molecular structure, chain length, functional groups, exposure duration, and treatment configuration. Importantly, the laboratory exposure levels reported in the literature frequently differ from concentrations measured in wastewater treatment plants. Moreover, some studies have reported limited effects at relatively high exposure levels, whereas pronounced effects may occur under prolonged exposure, highlighting the importance of reactor configuration, exposure duration, and biological endpoints when interpreting apparent concentration–response relationships.

## 5 Future prospects

Despite the growing body of evidence, direct comparisons among studies remain challenging because contaminant physicochemical properties, exposure concentrations, exposure durations, reactor configurations, wastewater matrices, and biological endpoints differ substantially. For nanomaterials, particle size, surface coating, aggregation state, dissolution, and surface reactivity can influence their bioavailability and biological effects. For microplastics, polymer type, particle size and shape, aging, surface characteristics, and associated additives contribute to variability among studies. For PFAS, chain length, functional groups, precursor transformation, and partitioning behavior can affect their environmental distribution and biological responses. In addition, many laboratory studies employ exposure levels and experimental conditions that differ from those encountered in wastewater treatment plants, while short-term batch experiments may not adequately represent chronic exposure under continuous operation. These differences introduce uncertainty when extrapolating laboratory findings to practical wastewater treatment systems and highlight the need for standardized exposure scenarios, longer-term experiments, and quantitative dose–response assessments. Therefore, their impacts, mechanisms of action, and recovery strategies for biological treatment systems urgently need to be systematically investigated from multiple perspectives and scales.

Firstly, attention should be given to the synergistic effects among pollutants. The interfacial adsorption, co-transport, and transformation mechanisms of composite systems—such as nanomaterials–antibiotics, microplastics–heavy metals, and PFAS–antibiotics—should be deeply analyzed in activated sludge and biofilms. A toxicity amplification network model should be established based on multi-omics data (metagenomics, metabolomics) and interfacial chemistry experiments to provide a basis for synergistic toxicity prediction using tools such as QSAR and machine learning models. Metagenomics can characterize microbial community composition and functional potential, metatranscriptomics can resolve changes in functional gene expression and stress responses, metaproteomics can determine whether predicted functions are translated into proteins, and metabolomics can link molecular responses to changes in cellular metabolic activity. Recent studies have demonstrated the value of these approaches in biological treatment systems. Metagenomic analysis of wastewater sludge exposed to gold nanoparticles has revealed changes in microbial communities associated with nanoparticle design, demonstrating the utility of community-level genomic analysis for resolving nanoparticle–microbe interactions (Metch et al., 2018). In addition, metagenomic–metatranscriptomic analysis of continuous PFOA exposure during anaerobic digestion showed that long-term exposure suppressed genes involved in glycolysis and pyruvate-to-acetyl-CoA conversion and was accompanied by reduced biogas production, illustrating how multi-omics approaches can connect molecular responses with process-level consequences (Besharati Fard et al., 2026). Evaluating the chronic effects of long-term, low-level exposure necessitates continuous-operation experiments over extended durations (six months to several years) to follow the accumulation, persistence, and transformation of pollutants such as short-chain PFAS and nanomaterial-derived degradation products. Intelligent online monitoring reactors should be constructed, using bio-indicator parameters such as EPS secretion characteristics, microbial community structure, and functional gene expression (e.g., amoA, dehalogenase genes) to enable real-time risk assessment and early warning.

For recovery strategies, multidisciplinary approaches should be promoted. Computational Fluid Dynamics simulations can reveal the diffusion and mass transfer pathways of pollutants in biofilms and flocs. Engineered strains designed via CRISPR/Cas9 and synthetic biology technologies can enhance the degradation and transformation of nanomaterials, microplastics, and PFAS (e.g., *E. coli* expressing metal-binding proteins or recombinant dehalogenase bacteria). In parallel, green nanomaterials (e.g., smart carriers mimicking diatom shells, cellulose-based nanocarriers) with ecological compatibility and controlled degradation properties should be developed and evaluated for their life-cycle ecotoxicity. Mechanistic models (e.g., ASM-PFAS extended model) can be combined with machine learning algorithms (e.g., random forest, deep learning) to quantify the influence of process parameters (SRT, DO, carbon and nitrogen source supplementation) on pollutant removal and microbial recovery, thereby constructing a dynamic optimization decision system.

Finally, considering the risk of transboundary transport and bioconcentration of PFAS, microplastics, and nanomaterials among different media (atmosphere, soil, groundwater), globally unified monitoring and evaluation standards covering typical derivatives, particle size ranges, and degradation products should be established. These standards can be combined with species sensitivity distribution models to conduct regional ecological risk assessments.

## 6 Conclusion

Nanomaterials, microplastics, and PFAS, as typical emerging contaminants, significantly disrupt the stability and microbial function of biological treatment systems due to their nanoscale effects, persistence, and complex coexistence. Their toxicity involves multiple pathways, including oxidative stress, gene expression interference, membrane disruption, and metabolic blockage, and the coexistence of pollutants may produce synergistic or antagonistic effects that further impair system performance. To mitigate these impacts, various strategies have been developed, including physical separation (membrane filtration, adsorption), chemical degradation (advanced oxidation, reduction reactions), and bioremediation (functional or engineered bacterial strains). Combined processes have shown initial success in improving pollutant removal, but require further optimization regarding cost, long-term stability, and secondary pollution risks.
